# Microfluidic Microcirculation Mimetic for Exploring Biophysical Mechanisms of Chemotherapy-Induced Metastasis

**DOI:** 10.3390/mi14091653

**Published:** 2023-08-22

**Authors:** Ashley Abraham, Sukhman Virdi, Nick Herrero, Israel Bryant, Chisom Nwakama, Megha Jacob, Gargee Khaparde, Destiny Jordan, Mackenzie McCuddin, Spencer McKinley, Adam Taylor, Conner Peeples, Andrew Ekpenyong

**Affiliations:** 1 Biology Department, Creighton University, Omaha, NE 68178, USA; ashleyabraham@creighton.edu (A.A.); nickherrero@creighton.edu (N.H.); meghajacob@creighton.edu (M.J.); gargeekhaparde@creighton.edu (G.K.); destinyjordan@creighton.edu (D.J.); mackenziemccuddin@creighton.edu (M.M.); spencermckinley@creighton.edu (S.M.); adamtaylor@creighton.edu (A.T.); 2 Physics Department, Creighton University, Omaha, NE 68178, USA; sukhmanvirdi@creighton.edu (S.V.); israelbryant@creighton.edu (I.B.); connerpeeples@creighton.edu (C.P.); 3 Chemistry Department, Creighton University, Omaha, NE 68178, USA; chisomnwakama@creighton.edu

**Keywords:** microfluidics, metastasis, cancer, mechanical properties, physics of cancer, chemotherapy, microcirculation, tumor microenvironment

## Abstract

There is rapidly emerging evidence from pre-clinical studies, patient samples and patient subpopulations that certain chemotherapeutics inadvertently produce prometastatic effects. Prior to this, we showed that doxorubicin and daunorubicin stiffen cells before causing cell death, predisposing the cells to clogging and extravasation, the latter being a step in metastasis. Here, we investigate which other anti-cancer drugs might have similar prometastatic effects by altering the biophysical properties of cells. We treated myelogenous (K562) leukemic cancer cells with the drugs nocodazole and hydroxyurea and then measured their mechanical properties using a microfluidic microcirculation mimetic (MMM) device, which mimics aspects of blood circulation and enables the measurement of cell mechanical properties via transit times through the device. We also quantified the morphological properties of cells to explore biophysical mechanisms underlying the MMM results. Results from MMM measurements show that nocodazole- and hydroxyurea-treated K562 cells exhibit significantly altered transit times. Nocodazole caused a significant (*p* < 0.01) increase in transit times, implying a stiffening of cells. This work shows the feasibility of using an MMM to explore possible biophysical mechanisms that might contribute to chemotherapy-induced metastasis. Our work also suggests cell mechanics as a therapeutic target for much needed antimetastatic strategies in general.

## 1. Introduction

Although most anti-cancer drugs target the proliferation of cancer cells, it is metastasis, the complex process by which cancer cells spread from the primary tumor to other tissues and organs of the body where they form new tumors, that leads to over 90% of cancer-related deaths [1,2,3,4]. Thus, there is an urgent need for anti-metastasis therapy alongside chemotherapy and radiotherapy [5,6,7,8]. An important step in the metastatic cascade is migration [9,10], which is connected with other crucial steps such as extravasation and intravasation. To migrate, cells actively alter their mechanical properties through their actin–myosin cytoskeleton [11]. We have recently shown in vitro [12] and others have shown in patient samples [5,7,13] that certain chemotherapeutic drugs inadvertently produce pro-metastatic effects. In this previous work [12], we did not explore the mechanisms behind these effects. Interestingly, following our work, several research groups reported that certain chemotherapeutics elicit a de novo prometastatic tumor microenvironment in both preclinical studies and in some patient subpopulations [14,15,16,17,18,19]. Expectedly, the very notion of chemotherapy-induced metastasis was initially disconcerting since chemotherapy is one of the main pillars of cancer treatment, but the aforementioned overwhelming evidence as well as newer clinical case reports [20] have not only led to a concerted search for the causal mechanisms but also to new therapeutic strategies aimed at mitigating chemotherapy-induced metastasis [21,22,23,24,25]. Some of the mechanisms of chemotherapy-induced metastasis reported include increase in extravasation and intravasation [15], increase in intravasation sites called tumor microenvironments of metastasis (TMEM) [16], increased migration and release of circulating tumor cells into the blood stream of cancer patients following chemotherapy [17], increased infiltration of immune cells such as neutrophils [18] and release of prometastatic proteins through extra-cellular vesicles [19]. Since most of these reported mechanisms involve migration, extravasation, intravasation and translocation, all of which are orchestrated by the mechanical properties of cells, a crucial question immediately presents itself: what is the role of cell mechanics in chemotherapy-induced metastasis? In other words, can cell mechanical properties orchestrate biophysical mechanisms of chemotherapy-induced metastasis?

In view of addressing this question, this work aims at showing the feasibility of using an MMM to investigate which chemotherapeutic drugs alter the mechanical properties of cancer cells in ways that might inadvertently promote metastasis before cell death. This investigation is at the heart of the physics of cancer, a new research frontier which explores the mechanical properties of cancer cells and their role in cancer disease and metastasis [10,26]. Fortunately, the role of cell mechanical properties in cancer metastasis in general has also become a focus of intensive research [27,28] giving further significance to our investigation. The question that we address here, namely, whether common anti-cancer drugs inadvertently change the cytoskeleton (mechanical makeup) of various cancer cells, thereby altering cell migration, extravasation and intravasation, posits the possibility of having cell mechanical properties as therapeutic targets in the context of the ongoing search for effective anti-metastasis drugs/protocols. Interestingly, our results show that chemotherapy-induced changes to the mechanical properties of leukemic cancer cells are drug-dependent. Nocodazole, an antineoplastic agent which arrests cells in the G2 or M phase and alters dynamic instability in microtubules [29], consistently induced a significant increase (*p* < 0.01) in transit times of K562 cells through the MMM, indicating increased stiffness. Hydroxyurea leads to significantly (*p* < 0.0001) reduced transit times. These results suggest new therapeutic strategies to mitigate metastases that might be induced by these drugs in the case of cancers that rely on their use. Moreover, in spite of the very wide variety of cancers with respect to their molecular biology, pathogenesis and prognosis, metastasis occurs in all cancers [4,30,31]. Thus, any contribution to anti-metastatic treatment strategies would be significant in the perennial and global fight against cancer.

Furthermore, using our simple but robust microfluidic and non-invasive tool to explore changes induced by chemotherapeutic drugs to the mechanical properties of a variety of cancer cells adds to the mechanomic library for other basic research and clinical investigations. Such a mechanomic library is useful beyond cancer research, being already envisaged in the intensely growing field of mechanical phenotyping for normal cell characterization and disease diagnosis [32,33,34]. The use of constriction-based microfluidic devices for the measurement of transit times and the characterization of single cell mechanical properties is well established and there are comprehensive reviews of these devices in the literature [35,36,37]. Most of the microfluidic devices of this category are made with polydimethylsiloxane, (PDMS) [38], just like our MMM. The adaptation of these devices for diagnostic applications based on single cell mechano-phenotyping are on the rise, including applications to malaria [39] and to end-stage kidney disease patients [40].

Succinctly, our work engenders innovations in three ways: novel mechanical characterization of various drug-treated cells to reveal impact of chemotherapy on cellular cytoskeleton, since several cell types have not yet been measured using the MMM with its unique mimicry of in vivo constrictions of the microvasculature; use of an inexpensive and yet robust microfluidic tool to posit the possibility of connections between cellular mechanical properties and cancer metastasis; and provision of scientific rationale for using cell biophysical properties as new therapeutic targets for more effective cancer treatment.

## 2. Materials and Methods

### 2.1. Microfluidics Microcirculation Mimetic, MMM

**Figure 1:**
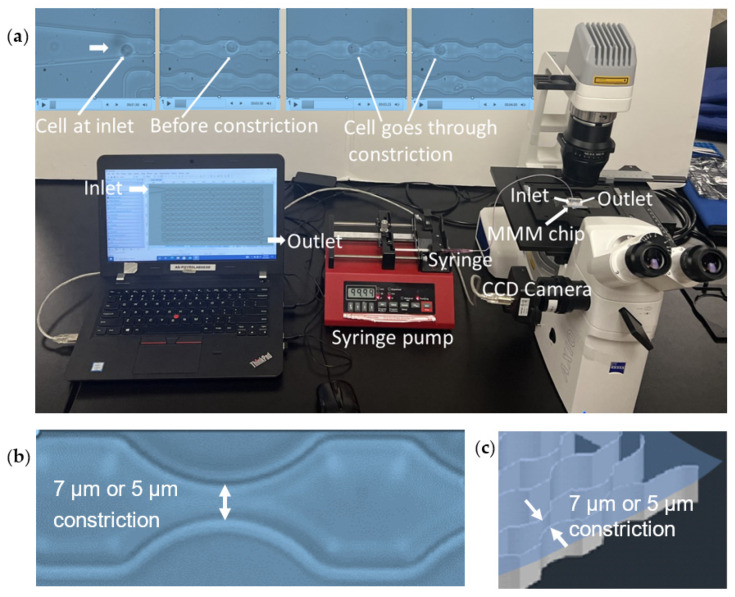
The microfluidic microcirculation mimetic (MMM). (**a**). A picture of the entire MMM device in operation. A suspension of cells diluted in PBS or culture medium in a syringe is fixed to a syringe pump, connected by tubing to the PDMS-based MMM chip inlet and placed on a phase contrast/fluorescence microscope with an inverted objective lens and equipped with a CCD camera. The camera is connected to a computer which enables the running and monitoring of the device, data collection and analysis. Four screengrabs from the video (top pictures from left) show a cell at the inlet, then before a constriction, into and out of a constriction, enabling the assessment of its deformability or mechanical properties, via its transit time through the device. (**b**). Picture showing the constriction size in 2D. (**c**). Schematic of 3D geometry showing the constriction size.

The MMM was developed as a microfluidic tool that mimics in vivo pulmonary microcirculation and has been extensively used for mechano-phenotyping [12,41,42,43,44]. The device mimics advection of cells in capillaries, many of which have constrictions smaller than blood cell diameters (Figure 1). This is also part of the circulatory phase of cancer metastasis. The MMM was developed in four main steps: (1) conceptualization/drawing using AutoCAD, (2) printing of photomask, (3) photolithography to produce master molds and (4) soft lithography for replica molding. Three variants of the MMM were made at the AutoCAD design phase: one without constrictions (constant width of 15 μm) and the others with 5 μm and 7 μm as the smallest constriction widths. All variants have a constant height of 15 μm. A polyester photomask of the design was printed commercially (Photo Data and J.D. Photo-Tools, JD Photo Data, Hitchin, UK). 

Using the photomask, the master molds were fabricated following standard photolithographic techniques (Institute of Semiconductors and Microsystems, Technische Universität Dresden, Germany). An upgraded design leading to 14 devices on a single master plate was developed (Potomac Photonics, Halethorpe, MD, USA). These master molds are used hundreds of times to make the MMM chips when needed, using soft lithography. The MMM was used to measure the mechanical properties of resting versus activated neutrophils in the context of chronic occlusive pulmonary disorder, COPD [41,45], with results corroborating those from a different device for mechanical phenotyping, namely, the optical stretcher [46,47]. The uniqueness of the MMM compared to other microfluidic devices used for mechano-phenotyping is highlighted in the description below.

The MMM is made using polydimethylsiloxane (PDMS). It consists of serpentine microchannels (Figure 1) that mimic the pulmonary microcirculation, with gently tapered inlet and outlet channels (see previous publications [12,41,43,44] for 3D schematics, in addition to the picture in Figure 1). The MMM differs from existing devices that model blood vessels in the lungs in that it does not involve branches or channel networks [48]. Rather, a single channel with constrictions in series is maintained for two reasons, namely, keeping the “view-point” of the cell and having each cell go through a large number of tractable constrictions. Firstly, from the “viewpoint” of the cell, constrictions occur one after the other, branches or no branches. Secondly, this serial model enables a very large number of constrictions (here, 187) to be within the field of view of the microscope objective. A constant pressure difference when using a pressure pump, or constant flowrate when using a syringe pump, is maintained between the inlet and the outlet, modeled on physiological pressure gradients experienced in vivo, and is used to advect cells through the device, one cell at a time. The minimum gaps at the constrictions (5 μm or 7 μm) are smaller than the diameter of cells but match the size of the pulmonary capillary segments, ensuring that each cell is deformed sequentially during the advection (Figure 1, top insets).

The MMM has been used for other research findings including the unravelling of some of the functions of myosin II [43]. It was the main tool used in one of the first reports of chemotherapy-induced prometastatic changes in leukemia cells (HL60 cells) [12], though the mechanisms behind the changes were not explored. Other clinically relevant research performed using an MMM (at least partially) includes the discovery that microgravity modulates effects of chemotherapeutic drugs on cancer cell migration [49]. Clinically, an MMM has been used for the ex vivo visualization of vaso-occlusive crises in sickle cell disease [44]. In summary, the biophysical properties of the following cell types in health and diseases have been measured using an MMM: neutrophils [41,45], leukemia cells [12], mesenchymal stromal cells [42] and red blood cells.

The MMM enables rapid measurement of hundreds of cells in less than 10 min, while watching in real time the fate of those cells during the advection, a step in metastasis, as would occur for circulating tumor cells (CTCs) [50,51]. Of note, metastasis is a complex multistep process that leads to death in over 90% of cancer cases [26,31]. Thus, the MMM mimics the hematogenic microcirculation in terms of attributes such as the presence of tens to hundreds of constrictions as found in microcapillary beds in vivo. Furthermore, to mimic the pluronan-rich surface of the vascular endothelium, Pluronic F-127 acid (final concentration 0.1%) was added to the cell suspension before the experiments [52]. Figure 1 illustrates the procedures for running the MMM: a suspension of cells in a syringe is placed on a programmable double channel microfluidics syringe pump, NE-4002X (MA, USA), connected by tubing to the polydimethylsiloxane-based MMM chip and placed on an inverted phase contrast microscope equipped with a CCD camera. The camera is connected to a computer which enables the monitoring and running of the device, data collection and analysis. We used the MMM for the determination of transit times, which is a readout of cell deformability [12,41].

### 2.2. Cell Culture

The K562 cells, human bone marrow-derived chronic myelogenous leukemia (CML) cells, were purchased from ATCC (Manassas, VA, USA) asK-562 ATCC^®^ CCL-243™, and grown in suspension using standard methods and media, namely, RPMI 1640, supplemented with 10% fetal bovine serum (FBS) and 1% penicillin/streptomycin. The cells were cultured in an incubator kept at 95% air, 5% CO_2_ and a temperature of 37 °C. We determined cell density using two methods: counting using a hemocytometer and using an automated cell counter (Invitrogen™ Countess™ II, ThermoFisher Scientific, Waltham, MA, USA, AMQAX1000). Cell viability was also determined in two ways: Trypan blue exclusion tests and fluorescence staining with calcein (Calcein AM, Invitrogen, ThermoFisher Scientific, Waltham, MA, USA, C1430). The cell density for all MMM experiments was maintained at about 1 × 10^6^ cells/mL to ensure that one cell went through the device before the next arrived at the inlet in general. Only cells at the logarithmic growth phase when viability was consistently over 96% were used for experiments.

### 2.3. Drug Treatments

Chemotherapeutic interventions were performed using common anti-tumor drugs, namely, nocodazole, hydroxyurea, doxorubicin and daunorubicin. Nocodazole is a cytoskeleton-changing drug and an antineoplastic agent which arrests cells in the G2 or M phase. It reversibly alters dynamic instability in microtubules [29]. Hydroxyurea is an antitumor and antileukemic agent (precisely CML) which functions by inhibiting mitosis, thereby blocking cell growth [53]. Doxorubicin, one of the most potent chemotherapeutic drugs, and daunorubicin, are anthracyclines which damage DNA in cancer cells and are used as first-line induction therapies against leukemias and many other cancers [54]. Cytochalasin D was used to disrupt the actin cytoskeleton of cells [11,47] in view of further exploration of the cellular architectures involved in deformability measurements via transit times through the MMM. All drugs were added to the cell culture medium for MMM experiments, and the cells were incubated for 10 min before starting MMM experiments, which were then completed at most 2 h after the drug treatment. Hence, no cell had the drug for more than 2 h in any MMM experiment reported here. This ensured that we were probing initial and potentially prometastatic effects of the drugs prior to cell-killing or other cytotoxic effects. Nocodazole was used at a 5 μM final concentration [55], while hydroxyurea was used at a 100 μM final concentration. The final concentration of doxorubicin was 5 μM [12,54], while that of daunorubicin was 1 μM [12,56]. Cytochalasin D was used at a final concentration of 2 µM [11,47].

### 2.4. Fluorometric Morphometry

Although morphometry has been performed on cells inside the MMM where shape parameters such as circularity and roundness were used to parameterize sickling of red blood cells in sickle cell disease patients [44] and fluorescently labeled neutrophils inside the MMM have been characterized morphometrically [41], we focused on just transit times of cells through the MMM in this work. However, fluorescence-guided morphometry independent of the MMM was performed using a Zeiss Vert.A1 AXIO fluorescence microscope (Carl Zeiss, Jena, Germany) equipped with red, green and blue filters and with an inserted USB microscope camera (AmScope MU300, Ningbo, China), along with its AmScope (86x) image capture program. Details of the advanced fluorometric morphometry performed which yielded information on cytoplasmic cross section and nuclear cross section, following chemotherapeutic interventions, can be found in our recent publications [57,58]. Briefly, the fluorescent dyes Hoechst 33342 (ThermoFisher Scientific, Waltham, MA, USA), at a 0.1 mM final concentration, and Calcein AM (Invitrogen by ThermoFisher Scientific, Waltham, MA, USA), at a final concentration of 1 µg/mL, were used to stain the nucleus and the cytoplasm, respectively, for imaging. ImageJ was used to extract cell shape and size, as well as nuclear shape and size, from the images. These morphometric measurements (size and shape), independent of MMM, serve to check whether differences in transit times are due to mechanical properties and not changes in cell size, shape or even adhesive properties.

### 2.5. Statistical Analysis

Analysis of variance, ANOVA, is a well-established statistical technique to analyze variations in continuous random variables measured under conditions defined by discrete factors [59] (parameters), thereby enabling the determination of the statistical significance of differences found in measured parameters. We performed statistical and error analyses using One-Way ANOVA in Origin (OriginLab, Northampton, MA, USA). We chose One-Way ANOVA or One-Way Fixed-Effects ANOVA because it is an extension of the Student 2-independent-samples *t* test and therefore enables one to simultaneously compare means among several independent samples [59]. Moreover, Origin’s ANOVA algorithm minimizes the probability of type-I error in statistical analysis, where the null hypothesis is wrongly rejected, leading to the wrong conclusion that results are statistically significant. For means comparison tests in Origin’s One-Way ANOVA, we selected the Tukey, Bonferroni, Dunn-Sidak and Fisher LSD, taking only those significant differences where all four tests were in agreement.

We performed error analysis using the standard error of the mean (SEM). For experiments where we expected to draw scientific conclusions, we carried out at least three independent repeats of every experiment (N1, N2 and N3). For exploratory experiments to show MMM capabilities, one experiment was conducted with sufficient cell numbers. In all experiments, information on cell numbers involved is provided.

## 3. Results

### 3.1. Nocodazole Increases Cell Transit Time

**Figure 2:**
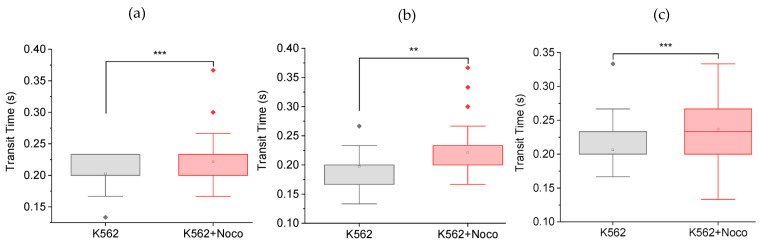
Nocodazole treatment increases cell transit times in MMM. Box plots from one-way ANOVA showing transit times of K562 cells and K562 cells treated for 2 h or less with 5 μM nocodazole (K562 + Noco). The box plots include outliers (filled diamond), mean (empty square) and shaded 75th to 25th percentiles (shaded rectangle). The 7 μm MMM device was used. Camera framerate: 30 fps. Pump flowrate: 99.9 μL/hr. This stiffening with nocodazole was consistent in all 3 repeats: N1, N2 and N3, shown in (**a**–**c**) respectively. (**a**) N1 experiment with K562 + Noco (*n* = 60 cells) showing a significantly higher (*** *p* < 0.001) mean transit time than untreated K562 cells (*n* = 60). (**b**) N2 experiment with K562 + Noco (*n* = 60 cells) showing a significantly higher (** *p* < 0.01) mean transit time than untreated K562 cells (*n* = 52). (**c**) N3 experiment with K562 + Noco (*n* = 60 cells) showing a significantly higher (*** *p* < 0.001) mean transit time than untreated K562 cells (*n* = 60).

We used the MMM as described in the Methods section to investigate whether nocodazole alters the mechanical properties of K562 cells in ways that might promote metastasis before cell death. Figure 2 shows that nocodazole treatment increases the transit times of cells in the MMM. Figure 2a shows a 10% increase in mean transit time from 0.203 ± 0.004 s to 0.222 ± 0.004 s between K562 cells and K562 cells treated for 2 h or less with 5 μM nocodazole (K562 + Noco). The box plot from one-way ANOVA shows that this increase in transit time is statistically significant (*** *p* < 0.001). Figure 2b,c are N2 and N3 repeats of the N1 experiment in Figure 2a. All three repeats lead to the same result, that nocodazole treatment increases mean cell transit times, which implies that nocodazole stiffens cells in the first 2 h of treatment. Since chemotherapy-treated cells in vivo include cells in the circulatory system, such stiffening or reduced deformability might increase the potential for cells to get stuck in the constrictions of the pulmonary vasculature, engendering an increased chance of extravasation and thus increased metastatic potential.

### 3.2. Feasibility of Exploring Biophyical Mechanisms

#### 3.2.1. Feasibility Testing Using Hydroxyurea

**Figure 3:**
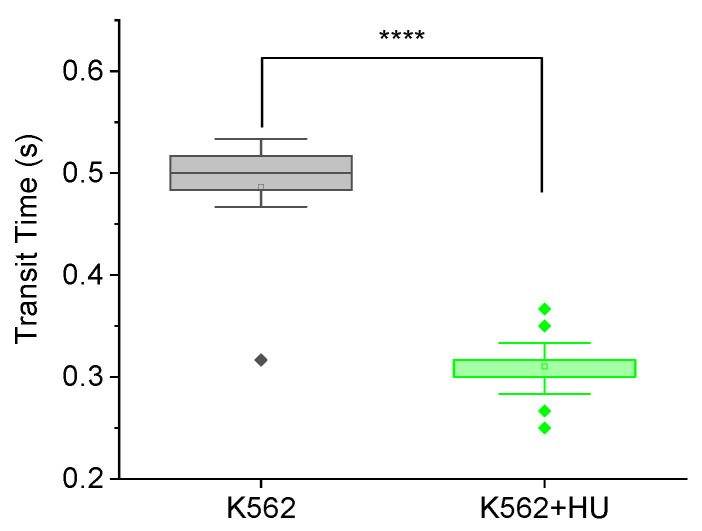
Feasibility of testing with hydroxyurea. Box plots showing transit times of K562 (*n* = 11) and K562 treated with hydroxyurea (K652 + HU, *n* = 60) through MMM. The box plots include outliers (filled diamond), mean (empty square) and shaded 75th to 25th percentiles (shaded rectangle). The 5 μm MMM device was used. Pump flowrate: 99.9 ul/hr. Camera framerate: 30 fps. The mean transit time of K562 + HU is significantly (**** *p* < 0.0001) lower than that of K562 cells.

Chemotherapeutic drugs have a wide variety of molecular mechanisms by which they act on various parts of the cell to produce cytotoxic effects. Aware of the differences in the mechanisms of action of nocodazole compared to hydroxyurea (for instance, they arrest cells in slightly different phases of the cell cycle [60]), we went on to explore the feasibility of deploying the MMM to pick up such differences in view of their potentially varied contributions to chemotherapy-induced metastasis. Remarkably, as shown in Figure 3, the mean transit time of K562 cells treated with hydroxyurea was significantly (**** *p* < 0.0001) lower than that of untreated K562 cells. Note that we used the more sensitive 5 μm MMM device here (and not the 7 μm MMM of Figure 2) so that its smaller constriction size might induce more mechanical stress on cells to produce more strain for picking up subtle mechanical differences. Figure 3 shows a 36% decrease in mean transit time from 0.486 ± 0.018 s to 0.310 ± 0.003 s between K562 cells and K562 cells treated with hydroxyurea, respectively. This result suggests that hydroxyurea treatment may increase cell deformability in the first two hours of treatment. This indicates the feasibility of deploying the MMM to explore various biophysical readouts that may correlate or even reveal mechanisms implicated in chemotherapy-induced metastasis.

#### 3.2.2. Size Effects: Independent Morphometry Tests

**Figure 4:**
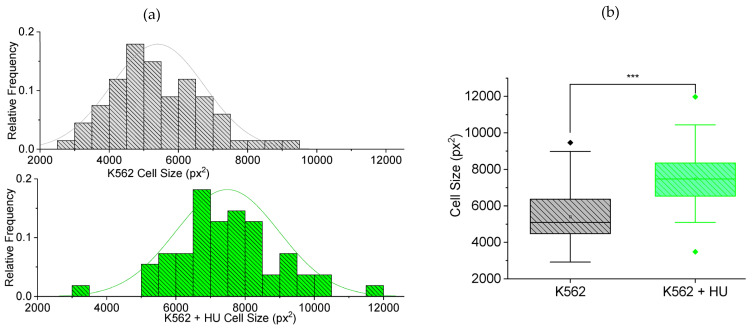
Fluorescence-guided morphometry to check effect on cell size, 12 h post hydroxyurea (HU) treatment. (**a**) Frequency histogram showing normally distributed cytoplasmic size (cell size) of K562 (*n* = 67) cells and a shift in the distribution towards larger sizes for K562 + HU (*n* = 55). (**b**) Box plots comparing the cells in (**a**), showing a significant (*** *p* < 0.001) increase in size of cytoplasm, suggesting cytoskeletal effects of HU treatment. The box plots include outliers (filled diamond), mean (empty square) and shaded 75th to 25th percentiles (shaded rectangle).

In our earlier work reporting how doxorubicin and daunorubicin alter cell mechanical properties in prometastatic ways [12], we monitored the morphology of cells at the time of drug treatment (0 h) and then 2, 4, 6 and 12 h after. It was only after 6 h with the drugs that cell sizes showed significant changes, consistent with known cytotoxic effects. Hence, in this work, we used fluorescence-guided morphometry to check effects on cell size, 12 h post hydroxyurea (HU) treatment. Figure 4a displays frequency histograms showing normally distributed cytoplasmic size of the cells. There is a shift in the distribution towards larger sizes for K562 treated with hydroxyurea. Figure 4b displays box plots comparing the cells in (a), showing a significant (*p* < 0.001) increase in size of cytoplasm. This result confirms that hydroxyurea treatment has some impact on the cytoplasm of cells 12 h post. Interestingly, the trend towards increasing cell size with drug treatment makes the reduced transit time of Figure 3 even more remarkable.

**Figure 5:**
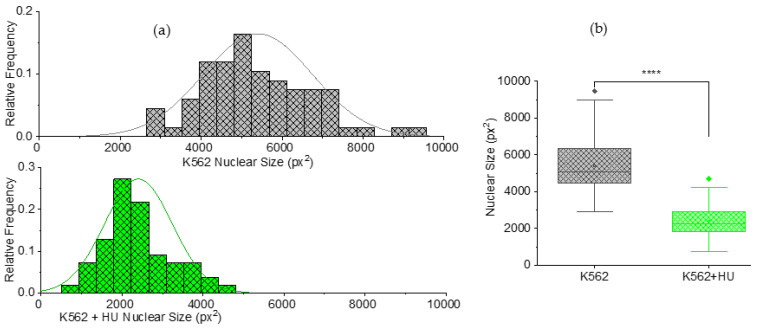
Fluorescence-guided morphometry to check effect on nuclear size, 12 h post hydroxyurea (HU) treatment. (**a**) Frequency histogram showing normally distributed nuclear size of K562 (*n* = 67) cells and a shift in the distribution towards smaller sizes for K562 + HU (*n* = 55). (**b**) Box plots comparing the cells in (**a**), showing a significant (**** *p* < 0.0001) decrease in size of nuclei due to HU treatment. The box plots include outliers (filled diamond), mean (empty square) and shaded 75th to 25th percentiles (shaded rectangle).

Furthermore, using the same fluorescence-guided morphometry as is performed for Figure 4, we extracted nuclear sizes from the cells in the experiment of Figure 4. The nuclear size results are presented in Figure 5 with frequency histograms showing normally distributed nuclear size (Figure 5a). There is a shift in the distribution towards smaller sizes for the hydroxyurea-treated cells. Figure 5b shows box plots comparing the cells in (a). Clearly, there is a significant (*p* < 0.001) decrease in size of nuclei due to hydroxyurea treatment.

Overall, changes in cell deformability, revealed by the MMM experiments and alterations in cytoplasmic and nuclear sizes shown via fluorescence-guided morphometry, are among biophysical properties modified by chemotherapeutic drugs. These properties can have an impact on cell migration, extravasation, intravasation and advection during circulation, all of which are important steps within the metastatic cascade.

### 3.3. Important Caveat: Unstable Framerates Can Lead to Inconsistent Results

**Figure 6:**
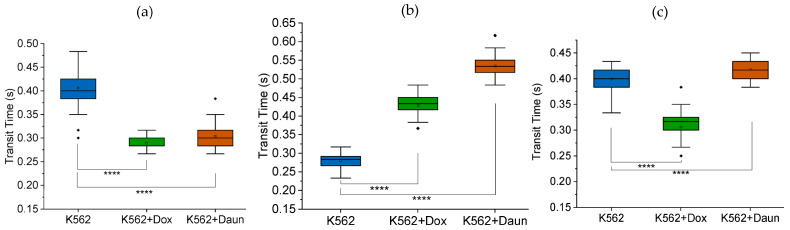
Unstable framerates lead to inconsistent results. Box charts from one-way ANOVA comparing transit time of K562 cells and K562 cells treated with doxorubicin (K562 + Dox) and daunorubicin (K562 + Daun). The box plots include outliers (filled diamond), mean (empty square) and shaded 75th to 25th percentiles (shaded rectangle). The 5 μm MMM device was used. Camera framerate: 60 fps. Pump flowrate: 99.9 μL/hr. The results were inconsistent due to unstable framerate when 60 fps was used: N1, N2 and N3, shown in (**a**–**c**), respectively, have different trends. In (**a**–**c**): K562 (*n* = 60 cells), K562 + Dox (*n* = 60 cells) and K562 + Daun (*n* = 60 cells), and **** *p* < 0.0001.

Obvious caveats for running microfluidic experiments accurately, or any scientific experiments, include generic verification that every equipment is functioning as expected. However, we deem it useful, as additional protocol hints, to show that fluctuating framerates, which may happen when higher framerates are chosen, may lead to inconsistent results. Figure 6 shows the inconsistent results we obtained when we changed from the stable framerate of 30 fps to 60 fps for our specific camera. The trends were inconsistent from N1 to N2 to N3. Even more convincing about the impact of framerates is Figure S1 where cytochalasin D, which disrupts F-actin and reduces the deformability of cells, showed inconsistent results due to fluctuations in framerates of 60 fps for our specific camera.

## 4. Discussion and Conclusions

Regarding our primary result that nocodazole increases cell transit time through the MMM, our interpretation that increased transit time implies increased stiffness is well supported in the literature. Using atomic force microscopy to measure cell stiffness and using fluorescence microscopy to visualize actin and microtubules, Grady et al. found that nocodazole-treated cancer cells exhibited increased stiffness [61]. Matching with our experimental conditions, Ito et al. found that nocodazole treatment for 60 min at a 10 μM final concentration rapidly stiffens cells [62]. Future experiments will be needed to illustrate the impact of nocodazole-induced stiffening of cancer cells on metastasis, along the lines of similar connections we found between doxorubicin-induced increase in transit time through the MMM and increased migration of HL60 cells via transwell migration assay [12]. Not all stiffening in the MMM should be taken as implying increased migration, just as we found in the case of daunorubicin where there was increased transit time in the MMM but reduced migration through pores in the transwell migration assay [12]. Different drugs and different cancer cells can be expected to show varied biophysical readouts that indicate complex mechanisms of chemotherapy-induced metastasis, just as metastasis itself has complicated and incompletely understood mechanisms. Our feasibility test with hydroxyurea illustrates this complexity. Recent work is cognizant of this complexity. For instance, Ly et al. [63] recently measured the deformability (via transit times through microfluidic constrictions) of B-cells that survive chemotherapy, taken from acute lymphoblastic leukemia (ALL) patients treated for 7 days with a standard multidrug chemotherapy regimen of vincristine, dexamethasone and L-asparaginase. They found that such B-cells are more deformable and distinguishable from controls (no chemo) [63]. They postulated, just as we do here, that cell physical phenotyping is a complementary prognostic tool that could guide treatment strategies and therapeutic interventions. Already, the mechanisms of chemotherapy-induced metastasis so far uncovered and new therapeutic strategies suggest the importance of biophysical aspects [21,22,23,24,25]. More concretely, multidrug chemotherapy regimens may have to include drugs that tune cell biophysical properties in antimetastatic ways.

Furthermore, our results in this work and the aforementioned corroborations in the literature suggest general connections between chemotherapy, cell mechanics and metastasis. Some aspects of these connections have been previously reported in the scientific literature. In fact, following recent realization that key stages in the metastatic cascade such as migration, extravasation and intravasation involve the mechanical and physical properties of cells [26], physical oncology or physics of cancer [10,26,64] has emerged, clearly illustrating the connection between cell mechanics and metastasis. Although chemotherapy drugs target and kill malignant cells during cancer treatment, there is emerging evidence that such drugs inadvertently promote metastasis [5,7,8]. The reported mechanisms of chemotherapy-induced metastasis [14,15,16,17,18,19] all involve migration, extravasation and intravasation which are orchestrated by the actin–myosin cytoskeleton [11], the same determinant of cell mechanical properties, clearly indicating the connection between cell mechanics and chemotherapy-induced metastasis. Furthermore, despite the very wide variety of cancers with respect to their molecular biology, pathogenesis and prognosis [65], metastasis occurs in all cancers, as noted already. Thus, there is an urgent need for anti-metastasis therapy [6] against all metastatic cancers, not just chemotherapy-induced metastasis. Incidentally, cellular mechanical properties have been shown to be good markers for metastatic potential of cancer cells [66,67] and can be used for diagnosis of cancer itself [9,34,68]. Hence, this work which explores the role of cell mechanics in chemotherapy-induced metastasis also addresses the role of cell mechanics in metastasis in general. Finally, it makes the case that cell mechanical properties should become therapeutic targets against both chemotherapy-induced metastasis as well as metastasis in general. This could lead to better treatment outcomes against cancers.

## Data Availability

Data are contained within the article and within Supplementary Material.

## Supplementary Materials

The following supporting information can be downloaded at: https://www.mdpi.com/article/10.3390/mi14091653/s1, Figure S1: Important caveats for running the MMM.
