# Supplementary material for: Microfluidic Microcirculation Mimetic for Exploring Biophysical Mechanisms of Chemotherapy-Induced Metastasis
Source: Micromachines (Basel). 2023 Aug 22;14(9):1653. doi: 10.3390/mi14091653 (PMC10536821; doi:10.3390/mi14091653)
Supplement: Supplementary file 1 [file micromachines-14-01653-s001.zip › micromachines-2514592-supplementary.pdf]

SUPPLEMENTARY INFORMATION

# Microfluidic Microcirculation Mimetic for Exploring Biophysical Mechanisms of Chemotherapy-Induced Metastasis

**Ashley Abraham, Sukhman Virdi, Nick Herrero, Israel Bryant, Chisom Nwakama, Megha Jacob, Gargee Khaparde, Destiny Jordan, Mackenzie McCuddin, Spencer McKinley, Adam Taylor, Conner Peeples, and Andrew Ekpenyong**

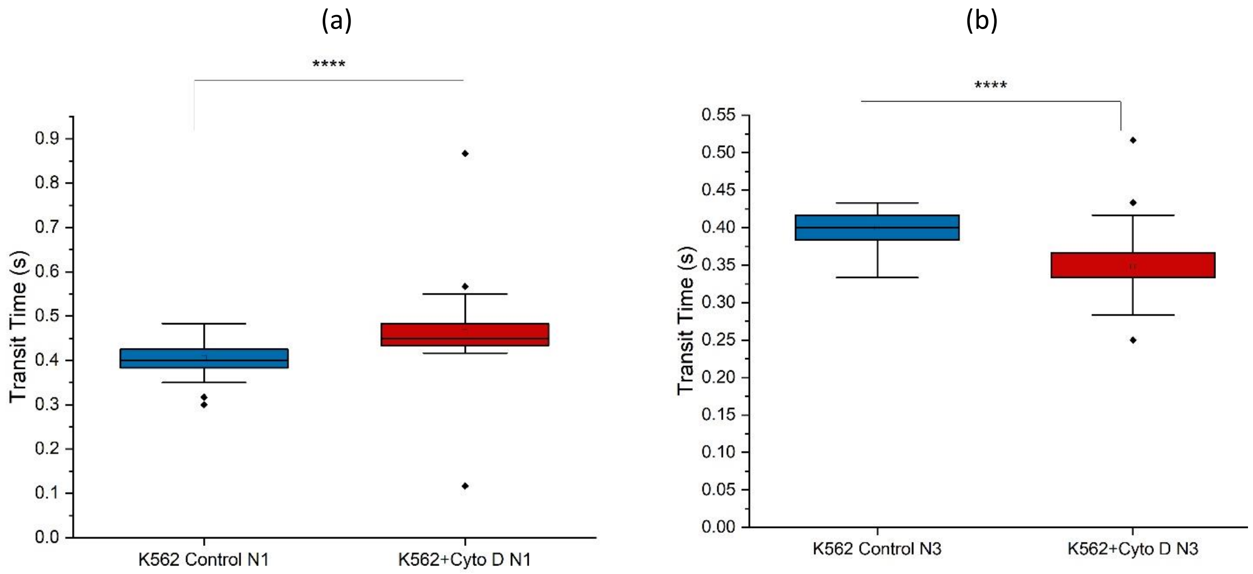


**Figure S1. Important caveats for running the MMM**. Inconsistent results may be obtained if camera frame rates become unstable. Camera frame rate, 60 fps (was unstable). MMM constriction size, 5 µm. Flow rate, 99.9 µl/hr. Box plots of transit times. (a) Experiment N1 shows that untreated cells (K562 Control) have significantly ($p < 0.0001$) **lower** transit times than those treated with cytochalasin D (K562+CytoD). (b) Experiment N3 shows that untreated cells (K562 Control) have significantly ($p < 0.0001$) **higher** transit times than those treated with cytochalasin D (K562+CytoD).
